# Valorization of Brewer’s Spent Grain Using Biological Treatments and its Application in Feeds for European Seabass (*Dicentrarchus labrax*)

**DOI:** 10.3389/fbioe.2022.732948

**Published:** 2022-05-03

**Authors:** Helena Fernandes, José Manuel Salgado, Marta Ferreira, Martina Vršanská, Nélson Fernandes, Carolina Castro, Aires Oliva-Teles, Helena Peres, Isabel Belo

**Affiliations:** ^1^ Departamento de Biologia, Faculdade de Ciências, Universidade Do Porto, Rua Do Campo Alegre Ed. FC4, Porto, Portugal; ^2^ Interdisciplinary Centre of Marine and Environmental Research (CIIMAR), Terminal de Cruzeiros Do Porto de Leixões, Av. General Norton de Matos, Matosinhos, Portugal; ^3^ Centre of Biological Engineering, University of Minho, Campus de Gualtar, Braga, Portugal; ^4^ Department of Chemistry and Biochemistry, Mendel University in Brno, Brno, Czech Republic

**Keywords:** solid-state fermentation, brewer’s spent grain, enzymatic hydrolysis, carbohydrases, aquaculture

## Abstract

Brewer’s spent grain (BSG) is the main brewery industry by-product, with potential applications in the feed and food industries due to its carbohydrate composition. In addition, the lignocellulosic nature of BSG makes it an adequate substrate for carbohydrases production. In this work, solid-state fermentation (SSF) of BSG was performed with *Aspergillus ibericus,* a non-mycotoxin producer fungus with a high capacity to hydrolyze the lignocellulosic matrix of the agro-industrial by-products. SSF was performed at different scales to produce a crude extract rich in cellulase and xylanase. The potential of the crude extract was tested in two different applications: -(1) - the enzymatic hydrolysis of the fermented BSG and (2) - as a supplement in aquafeeds. SSF of BSG increased the protein content from 25% to 29% (w/w), while the fiber content was reduced to 43%, and cellulose and hemicellulose contents were markedly reduced to around 15%. The scale-up of SSF from 10 g of dry BSG in flasks to 50 g or 400 g in tray-type bioreactors increased 55% and 25% production of cellulase and xylanase, up to 323 and 1073 U g^−1^ BSG, respectively. The optimum temperature and pH of maximal activities were found to be 55°C and pH 4.4 for xylanase and 50°C and pH 3.9 for cellulase, cellulase being more thermostable than xylanase when exposed at temperatures from 45°C to 60°C. A Box–Behnken factorial design was applied to optimize the hydrolysis of the fermented BSG by crude extract. The crude extract load was a significant factor in sugars release, highlighting the role of hydrolytic enzymes, while the load of fermented BSG, and addition of a commercial β-glucosidase were responsible for the highest phenolic compounds and antioxidant activity release. The lyophilized crude extract (12,400 and 1050 U g^−1^ lyophilized extract of xylanase and cellulase, respectively) was also tested as an enzyme supplement in aquafeed for European seabass (*Dicentrarchus labrax*) juveniles. The dietary supplementation with the crude extract significantly improved feed and protein utilization. The processing of BSG using biological treatments, such as SSF with *A. ibericus,* led to the production of a nutritionally enriched BSG and a crude extract with highly efficient carbohydrases capable of hydrolyzing lignocellulosic substrates, such as BSG, and with the potential to be used as feed enzymes with remarkable results in improving feed utilization of an important aquaculture fish species.

## Introduction

The brewery industry produces millions of tons of residues, arising environmental and ecological concerns (Farcas et al., 2017). Brewer’s spent grain (BSG) is an insoluble material comprising barley grain husks, small parts of the pericarp, and seed coat layers of the grains (Mussatto, 2014), representing 85% of the total residues originated from the brewing industry (Nocente et al., 2019). BSG is a lignocellulosic material mainly constituted by arabinoxylan, cellulose, and lignin, which can be degraded by hydrothermal, enzymatic, or acidic hydrolysis (Farcas et al., 2017). The protein content of BSG varies depending on the type of barley or other cereal mixture and the conditions applied during wort production (Mussatto, 2014; Farcas et al., 2017). BSG also presents phenolic compounds, such as ferulic and *p*-coumaric acids, with antioxidant properties (Aliyu & Bala, 2013), mainly associated with sugars, organic acids, lipids, and amines, limiting their bioavailability and antioxidant capacity (Dulf et al., 2016). BSG can be utilized for the production of a wide range of enzymes, such as carbohydrases, proteases, and laccases (Mussatto, 2014). Moreover, BSG can be used to obtain other value-added products, such as phenolic compounds (Birsan et al., 2019; Alonso-Riaño et al., 2020), lactic acid (Mussatto, 2014), and fermentable sugars (Paz et al., 2019). Concomitantly, BSG has numerous applications in the food industry (Canedo et al., 2016; Nocente et al., 2019), animal nutrition (Getu et al., 2020; San Martin et al., 2020), and in the production of biofuels (Dudek et al., 2019; Hakobyan et al., 2020). However, to enhance the BSG nutritional value and obtain high-valued compounds, such as protein, enzymes, and phenolic compounds, BSG may undergo biotechnological processes, such as solid-state fermentation (SSF).

SSF is a biotechnological procedure that adds value to the economically uninteresting agro-industrial by-products by promoting the growth of selected microorganisms, using these by-products as physical and nutritional support. During the SSF of lignocellulosic substrates, filamentous fungi partially degrade the vegetable matrix by producing carbohydrate-degrading enzymes (carbohydrases), increasing the release of matrix-linked phenolic compounds. SSF can be used as a pretreatment to enhance the fermentable sugar yield by promoting the lignocellulosic cell-wall disruption (Fernandes et al., 2019) or obtain a nutritionally enhanced biomass or a bioactive extract with, for example, antioxidant and enzymatic properties, without resorting to hazardous chemicals for the extraction (Martins et al., 2011). Ascomycetes fungi are suitable for the SSF of a wide range of lignocellulosic-rich substrates due to their ability to produce enzymes that degrade the complex lignocellulosic matrix (Ferreira et al., 2016). Among the Ascomycetes fungi, *Aspergillus* spp. has been extensively used to produce different value-added compounds, such as organic acids, chitosan, and a wide range of enzymes, including high levels of carbohydrases (Ferreira et al., 2016).

For the monogastric animal feed industry, including aquafeeds, the utilization of carbohydrases is utterly important, given their potential to enhance the digestibility of non-starch polysaccharides (NSPs) present in plant feedstuffs, such as cellulose and hemicellulose, which are indigestible for monogastric animals. In aquafeeds, the utilization of extracts rich in enzymes and/or phenolic compounds with antioxidant properties derived from the SSF of the agro-industrial by-products has been applied with promising results. For instance, Novelli et al. (2017) observed that incorporating fungal phytase and protease produced by SSF of the agro-industrial by-products with *A. oryzae* and *A. niger*, respectively, improved the dry matter, protein, lipid, and energy digestibility in Nile tilapia (*Oreochromis niloticus*). A commercial enzymatic mixture obtained from the SSF with *A. niger* (Synergen™, Alltech, United States) significantly improved feed utilization and growth of Nile tilapia (Bowyer et al., 2020). Including 0.1% Synergen™ in diets with white lupin meal enhanced the growth and feed efficiency of common carp (*Cyprinus carpio*) (Anwar et al., 2020). These enzymatic supplements have shown to be highly efficient in improving fish feed utilization with high environmental and economic relevance for the long-term sustainability of aquaculture and marine ecosystems.

**FIGURE 1:**
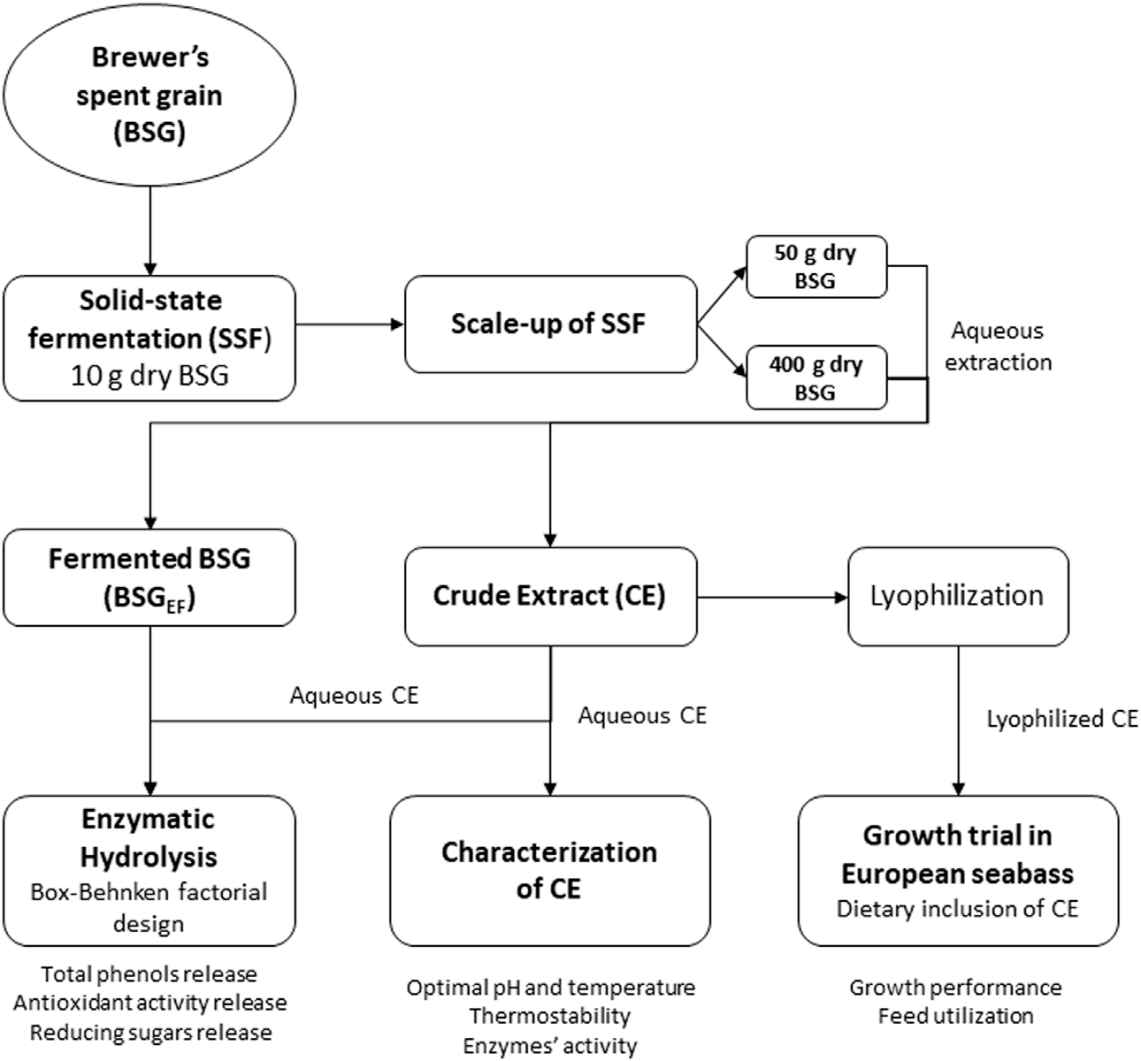
Schematic diagram of the production of a crude extract from solid-state fermentation of brewer’s spent grain with *A. ibericus* and its application to further hydrolyze the fermented BSG and as an aquafeed enzyme supplement.

It was previously observed that *A. ibericus* produced xylanase (313.8 U g^−1^), cellulase (51.3 U g^−1^), and β-glucosidase (4.06 U g^−1^) during SSF of BSG (Leite et al., 2019). The present study aimed to scale up the production of these enzymes and tested them as a novel functional additive to aquafeeds. In the first step, BSG was fermented by *A. ibericus* for hydrolytic enzyme production. This crude enzymatic extract was characterized by its thermostability, optimum pH, and temperature. In a second step, this crude extract was used to hydrolyze the remaining fermented BSG, and the optimum conditions, within the range of values defined for each variable, to maximize sugars and phenolic compounds release were determined. To validate the application of this extract as an aquafeed enzyme supplement, a feeding trial was carried out to assess its effect on growth performance and feed utilization efficiency of European seabass (*Dicentrarchus labrax*) juveniles. A schematic diagram of the process is presented in Figure 1.

## Materials and Methods

### Ethical Approval

This study was approved by the ORBEA Animal Welfare Committee of CIIMAR and the Portuguese National Authority for Animal Health (DGAV). Experiments were directed by trained scientists (Functions A, B, C, and D defined in article 23 of the European Union Directive 2010/63) and conducted following the Federation of Laboratory Animal Science Association (FELASA) recommendations and the EU Directive (2010/63/EU) on the protection of animals for scientific purposes.

### BSG and Selected Microorganism

BSG, provided by *Unicer*, Porto, Portugal, a soft-drink company, was constituted by Pilsner malt, barley, and corn gritz. The fungus species used in the SSF was *Aspergillus ibericus* (MUM 03.49), isolated from wine grapes (Serra et al., 2006). The fungus was maintained in potato dextrose agar at 4°C until utilization.

### Solid-State Fermentation of BSG and Production of the Crude Extract

The SSF was performed in duplicate in 500 ml cotton-plugged Erlenmeyer flasks with 10 g of dry BSG without nutritional supplements. BSG was sterilized at 121°C for 15 min and then inoculated with a spore’s suspension of *A. ibericus* (2 × 10^6^ spores/g dry BSG) following the optimized SSF of BSG described by Leite et al. (2019). Initial moisture of BSG was around 70% but was adjusted to 75% (w/w, wet basis) with the inoculum suspension and distilled water if needed. The height of the SSF bed in flasks was 1.5 cm. Each Erlenmeyer flask was then incubated at 25°C for 7 days. The approximate composition of BSG was determined before and after SSF, as present in Table 1.

The scale-up of SSF was performed in trays (16 x 11 x 6 cm or 43 x 33 x 7 cm), with 50 g or 400 g of BSG, in duplicate and triplicate, respectively, without additional nutrients. In each tray, the bed height was adjusted to 2.5 cm and the moisture level was adjusted to 75% with distilled water, followed by sterilization at 121°C for 15 min. Each tray was inoculated with a spore’s suspension with 2 × 10^6^ spores/g dry BSG, covered with perforated plastic wrap, and was incubated at 25°C for 7 days.

After the SSF, the fermented BSG was subjected to aqueous extraction with distilled water (1:5, g BSG: ml of water) for 30 min, with continuous agitation, at room temperature, followed by filtration through a nylon net (particle size <0.1 mm). The crude extract was obtained after the centrifugation (4,000 *g*, 15 min) of the liquid phase, separating the crude extract from the fungal biomass in suspension. A part of the crude extract was stored at −20°C for cellulase and xylanase activity analysis and the Box–Behnken factorial design experiments, and the other part was lyophilized to allow its incorporation in the fish diets. The extracted and fermented solid remaining after filtration (BSG_EF_) and the fungal biomass remaining after centrifugation were dried at 50°C, weighed, and stored at room temperature to be characterized.

### Optimum Temperature and pH for Xylanase and Cellulase Activities of the Crude Extract

To study the effect of temperature on xylanase and cellulase activities of the crude extract, the enzymatic activities were measured at temperatures ranging from 30°C to 70°C, with 5°C intervals, at a constant pH of 4.8. To assess the effect of the pH on these enzymes, the activities were measured at a constant temperature of 50°C and pH ranging from 2.6 to 5.8, and using sodium citrate 0.05 N as a buffer.

### Thermostability of Cellulase and Xylanase of the Crude Extract

The thermostability of cellulase and xylanase of the crude extract was determined by measuring the enzymatic activities after incubation at 45°C, 50°C, and 60°C for 120 min. The enzymatic activity assay was then performed at standard conditions (temperature of 50°C, pH 4.8) and expressed as a percentage of relative activity respectively to the initial value.

### Enzymatic Hydrolysis of BSG_EF_

An incomplete Box–Behnken design was performed to determine the optimal conditions for the hydrolysis of BSG_EF_ within the experimental levels used in each variable, aiming to maximize antioxidant activity potential and saccharification. The Box–Behnken design was performed with three factors at three levels (-1, 0, and +1), namely, the BSG_EF_ load (% w/v), crude extract amount (expressed as U cellulase g^−1^ of dry BSG_EF_), and commercial β-glucosidase amount (expressed as U g^−1^ of dry BSG_EF_). The design was performed in a set of 15 experiments, and three central point replicates were used to estimate the experimental error. For statistical calculations, the independent variables were coded, and the correspondence between the coded and uncoded variables is shown in Table 2. The dependent variables were the maximum values of total phenols, antioxidant activity, and reducing sugars released after enzymatic hydrolysis.

**TABLE 1:** Proximal composition of BSG before and after the SSF by *A. ibericus* in 500-ml cotton-plugged Erlenmeyer flasks followed by aqueous extraction (mean ± standard deviation)^*^ and percentage of mass variation of each component.

Compound (% w/w dry basis)	Unfermented BSG	BSG_EF_	Mass variation (%)
Protein	25.30 ± 0.02^a^	29.31 ± 0.31^b^	25
Fiber	59.94 ± 2.01^b^	43.30 ± 0.30^a^	53
Cellulose	21.16 ± 0.99^b^	14.73 ± 0.39^a^	55
Hemicellulose	23.77 ± 1.4^b^	15.29 ± 0.2^a^	58
Lignin	15.01 ± 0.41^a^	13.28 ± 0.72^a^	42

$\text{\% Mass variation}=\frac{\left({\left(\text{\%w}/\text{w}\right)}_{\text{unfermented}}x10\text{ g}-{\left(\text{\%w}/\text{w}\right)}_{\text{fermented}}x6.45\text{g}\right)}{{\left(\text{\%w}/\text{w}\right)}_{\text{unfermented}}x10\text{ g}}$ .

* Means in the same row and different superscript letters are significantly different (Tukey's test, p<0.05).

The Box–Behnken design experiments were performed as follows: BSG_EF_ was submitted to enzymatic hydrolysis with the crude extract and/or a commercial β-glucosidase (from *Aspergillus niger*, Megazyme; E-AMGDF). For that purpose, BSG_EF_ was mixed with sodium citrate buffer (0.05 N, pH 3.9), autoclaved for 15 min at 121°C, and mixed with the crude extract and/or commercial β-glucosidase. Thymol (0.07 g/l) was added to avoid contamination during hydrolysis. The mixtures were then incubated for 72 h at 45 °C with constant shaking (150 rpm). At the end of the enzymatic hydrolysis (72 h), the liquid was separated from the solid fraction by vacuum filtration, and the liquid fraction was stored at −20 °C for analysis of phenolic compounds, antioxidant activity, and free reducing sugars.

Based on the Box–Behnken design results, the best conditions predicted by the model within the levels studied for each variable to maximize the sugars and phenolic compounds released during the hydrolysis of BSG_EF_ with crude extract were tested in duplicate.

### Effect of Dietary Supplementation With the Crude Extract in European Seabass Growth and Feed Efficiency

To test the potential of the crude extract as an exogenous enzyme supplement for aquafeeds, a growth trial with a carnivorous aquaculture species, European seabass (*Dicentrarchus labrax*), was performed. For that purpose, three isoproteic (48% crude protein) and isolipidic (16% crude lipids) diets were formulated, containing 10% of fish meal and 60% of plant feedstuffs (% diet) as main protein sources, and fish oil as the main lipid source. The lyophilized crude extract (12,400 and 1050 U g^−1^ of xylanase and cellulase, respectively) was incorporated in the diets (w/w) at increasing levels of 0 (control diet), 0.1% (CE0.1 diet), and 0.4% (CE0.4 diet), corresponding to around 0 U, 1000 U, and 4000 U of cellulase g^−1^ diet (dry matter basis).

The growth trial was conducted in a thermoregulated recirculating water system, equipped with nine fiberglass tanks of 60 l capacity. The fish were obtained from a commercial aquaculture facility and acclimatized to the experimental system conditions for 15 days. Then, nine groups of 15 fish with an initial body weight of 22 g were established and randomly assigned to each tank. Triplicate groups were fed each experimental diet, by hand, until apparent satiety, twice a day, 6 days per week, for 64 days. Feed consumption was recorded weekly. During the trial, the water temperature was maintained at 23.8 ± 0.1°C, salinity at 32.0 ± 0.9‰, and nitrites and ammonia levels were kept below 0.05 mg ml^−1^. The water flow in each tank was kept at 5 L min^−1^. At the beginning and end of the experiment, fish were bulk-weighed after 1 day of feed deprivation to determine the growth and feed utilization parameters, namely, final body weight, weight gain, daily growth index, feed intake, feed efficiency, and protein efficiency ratio. Five fish from the initial stock and from each tank at the end of the trial were sampled and pooled for the whole-body composition analysis to measure the nitrogen and energy retention.

### Analytical Methods

#### Protein and Lignocellulosic Composition of Unfermented BSG and BSG_EF_

The protein content of the unfermented BSG and BSG_EF_ was assessed by the *Kjeldahl* method after the digestion with sulfuric acid (>95%) using a *Kjetelc* system (Foss 8400) and applying the factor 6.25 to convert N to protein. The lignocellulosic characterization of the unfermented BSG and BSG_EF_ was carried out following the method described by Leite et al. (2016).

#### Enzyme Activity Determination

Cellulase, xylanase, and β-glucosidase activities were measured in the crude extract. Cellulase activity was measured using carboxymethyl cellulose (2% w/v) as the substrate in sodium citrate buffer 0.05 N (pH 4.8), incubated at 50°C for 30 min, and the reducing sugars measured by the DNS method. Xylanase activity was determined using xylan (1% w/v) as substrate in sodium citrate buffer 0.05 N (pH 4.8), incubated at 50°C for 15 min, and the liberated reducing sugars measured by the DNS method (Miller, 1959). β-glucosidase activity was determined in accordance with the method by Leite et al. (2016) using β-d-glucopyranoside (pNPG) as substrate in acetate buffer (50 mM, pH 5).

One unit (U) of enzyme activity was defined as the enzyme quantity necessary to release 1 µmol of xylose/min or 1 µmol glucose/min from each substrate for xylanase or cellulase activities, respectively, and to release 1 μmol/min of *p*-nitrophenol for β-glucosidase activity. The enzymatic activities were expressed as U per gram of dry BSG or per gram of lyophilized crude extract (U g^−1^).

#### Total Phenols, Antioxidant Activity, and Reducing Sugar Analysis

After the enzymatic hydrolysis of BSG_EF_, total phenol (TP) content was determined by the Folin–Ciocalteau method, using gallic acid as standard, and the results were expressed in mg gallic acid equivalents (GAE)/g BSG_EF_. The antioxidant activity (AA) was analyzed by the DPPH method, as described by Fernandes et al. (2019), and the results were expressed in µmol Trolox equivalents (TE)/g BSG_EF_. The released reducing sugars (glucose, xylose, and arabinose) were analyzed by high-performance liquid chromatography using a Jasco830-IR intelligent refractive-index detector with a Varian MetaCarb 87H column. The column was eluted with 0.005 M H_2_SO_4,_ and the flux was settled at 0.7 ml min^−1^ at 60°C. The results for each sugar and total released reducing sugars (sum of glucose, xylose, and arabinose) were expressed as mg g^−1^ BSG_EF_.

### Statistical Analysis

The Box–Behnken experimental design data were evaluated using the response surface methodology by *Statistica 10* software (Informer Technologies Inc., LA, United States), and the dependent variables were optimized using the *Solver* tool (Microsoft Excel 2019; Redmon, WA, United States). The statistical analysis of data was performed by one-way analysis of variance (ANOVA), and Tukey’s multiple range test was used to detect significant differences among means (*p* < 0.05).

*StatgraphicPlus Centurion XVI* (Statgraphics Technologies Inc., Virginia, United States) was used to evaluate the data of the Box–Behnken experimental design, and *IBM SPSS Statistics 26* (IBM, NY, United States) was used for the data of the growth trial with the European seabass.

## Results

### BSG Fermentation

The fungi *A. ibericus* grew well in wet BSG at the used operational conditions, and fungal dry biomass of 1.8% (w/w, per dry mass of unfermented BSG) was produced.

At the end of the small-scale SSF using 10 g BSG in flasks, it was possible to recover 6.45 g of dry fermented BSG after aqueous extraction, corresponding to a total mass decrease of 35.5%. This BSG mass loss was due to the solubilization of BSG components that occurred through the SSF, resulting in a total protein mass decrease of 25%. Nevertheless, BSG_EF_ has a higher content of protein (29.3%, w/w) and a lower content of lignocellulosic fiber than the unfermented BSG (Table 1). The fiber content of BSG_EF_ was reduced by 53% relative to the unfermented BSG, corresponding to 58% hemicellulose, 55% cellulose, and 42% lignin degradation.

### SSF Scale-Up and Crude Extract Production

**FIGURE 2:**
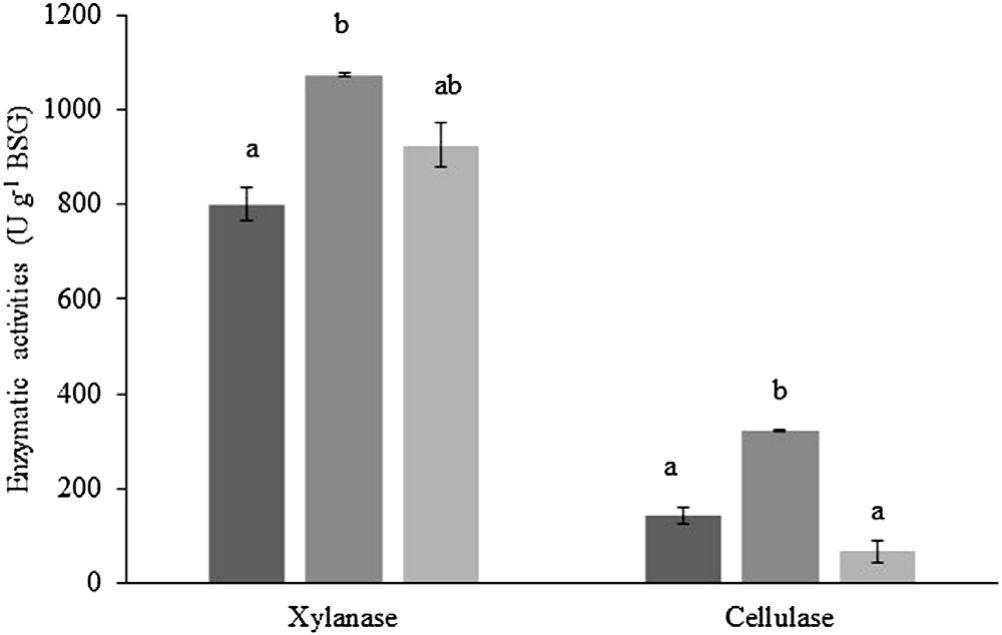
Xylanase and cellulase activities obtained by the SSF at different scales (black—10 g, dark gray—50 g, and light gray—400 g of dry BSG). Treatments without a common letter are statistically different (Tukey’s test; *p* < 0.05). Values are presented as the mean ± standard deviation of two (10 g and 50 g of dry BSG) and three (400 g of dry BSG) independent replicates.

Xylanase and cellulase production throughout the scale-up process of SSF of BSG, without nutrient supplementation, is presented in Figure 2. Values for β-glucosidase activity are not shown, given its low activity; however, the highest β-glucosidase activity (5.3 ± 0.3 U g^−1^ BSG) was achieved using 50 g of BSG in SSF. Maximum xylanase (1,072.9 ± 4.2 U g^−1^ BSG) and cellulase (323.2 ± 0.9 U g^−1^ BSG) activities were, in both cases, obtained by the SSF of 50 g of BSG, corresponding to an activity increase of 25% and 55%, respectively, relative to the SSF of 10 g of BSG. Further increase in the quantity of BSG subjected to SSF, up to 400 g, slightly (14%) decreased xylanase activity without statistical significance, but cellulase activity was significantly reduced by 79%. However, the enzyme activities obtained with 10 g and 400 g of BSG (40-fold scale-up) were not statistically different.

### Optimum Temperature and pH for the Crude Extract Xylanase and Cellulase Activity

**FIGURE 3:**
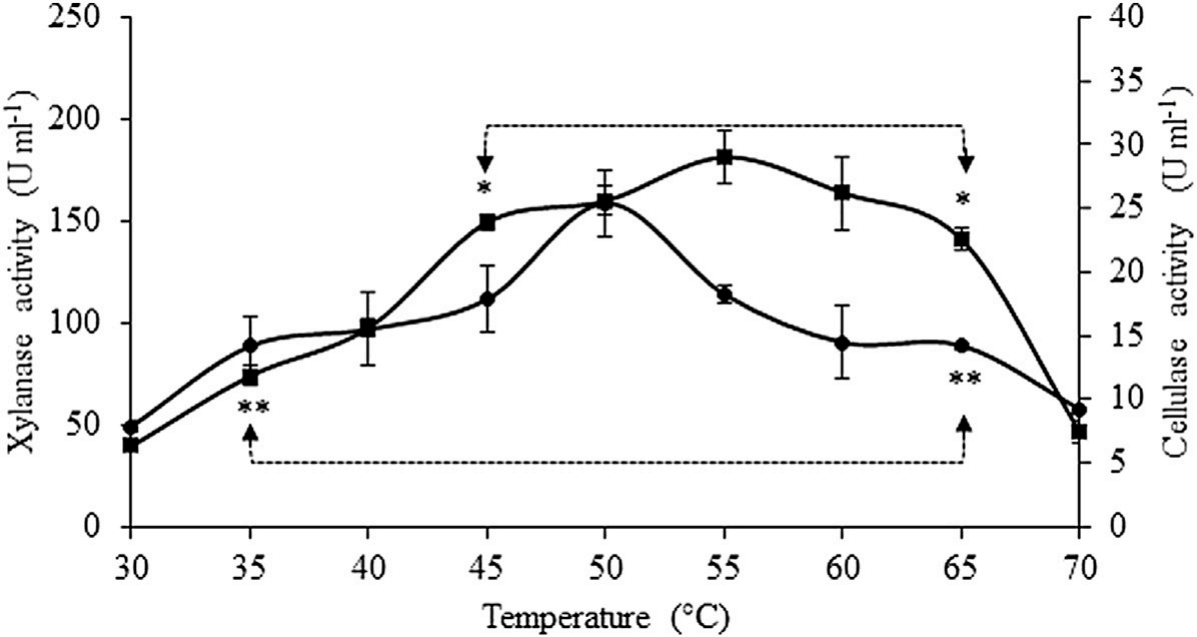
Effect of temperature on xylanase (■) and cellulase (•) activities. Asterisks represent the interval of temperatures and pH in which no statistical differences were detected (Tukey’s test, *p* < 0.05) for xylanase (*) and cellulase activities (**). Values are presented as the mean ± standard deviation of two independent replicates.

The effect of temperature on xylanase and cellulase activities is presented in Figure 3. Xylanase activity significantly increased (*p* < 0.05) from 30°C to 45°C (3.8-fold), with its optimal range of temperature observed between 45°C and 65° C and a maximum activity at 55° C (181.5 ± 13.1 U ml^−1^ crude extract), without significant statistical differences among those values. Above 65° C, a significant drop of 67% in xylanase activity was observed when the reactional temperature was 70° C. Maximum cellulase activity was attained at 50° C (25.4 ± 2.6 U mL^−1^ crude extract), but no statistically significant differences (*p* < 0.05) were found for cellulase activities in the temperatures ranging between 35°C and 65° C. The lowest values of cellulase activity were found at 30°C and 70° C, being 69% and 64% lower, respectively, than the those observed at 50°C.

**FIGURE 4:**
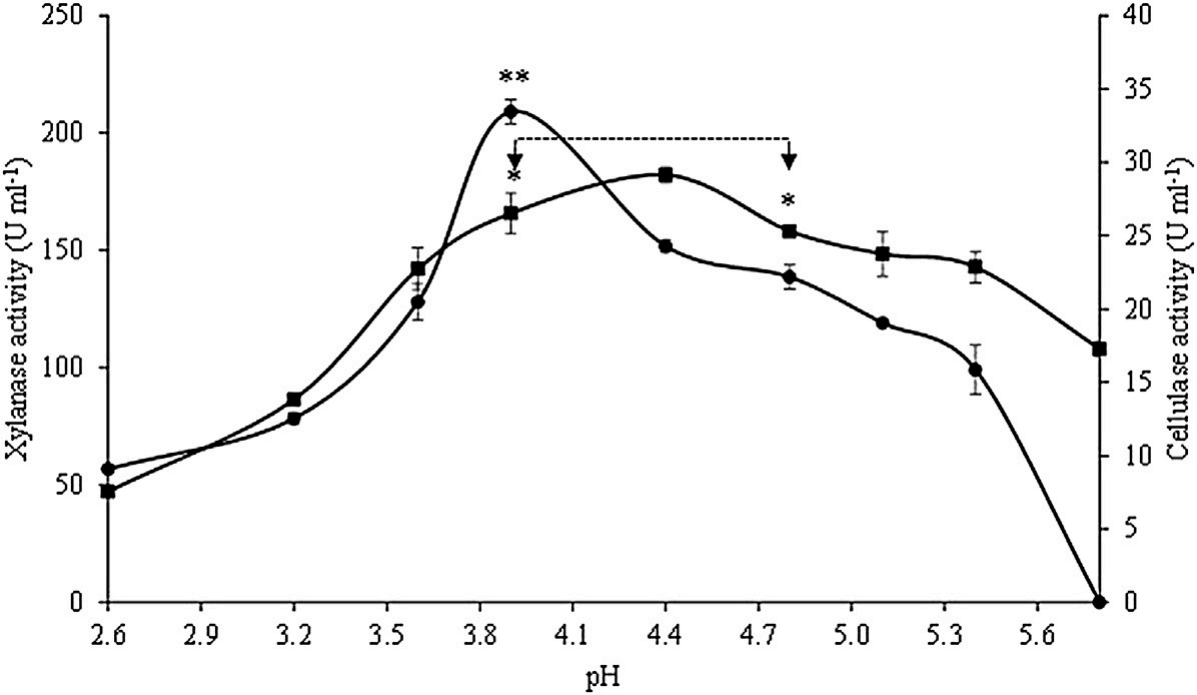
Effect of pH on xylanase (■) and cellulase (•) activities. Asterisks represent the interval of temperatures and pH in which no statistical differences were detected (Tukey’s test, *p* < 0.05) for xylanase (*) and cellulase activities (**). Values are presented as the mean ± standard deviation of two independent replicates.

The effects of pH on the crude extract xylanase and cellulase activity are presented in Figure 4. Xylanase activity was the highest at pH ranging between 3.9 and 4.8, with maximum activity (182.2 ± 3.4 U ml^−1^ crude extract) at pH 4.4, without significant statistical differences (*p* < 0.05) among the activities within this pH range. High levels of xylanase activity were still observed at pH ranging from 3.6 to 5.4, with activity values above 78% of the maximum value. At the lowest pH studied, xylanase activity decreased 74%, while the activity only decreased 41% at the highest pH. Optimum cellulase activity was observed at pH 3.9 (33.5 ± 0.8 U ml^−1^ crude extract), and this value was statistically different from the values obtained at all the other pH conditions tested. The cellulase activity in the crude extract was above 61% of its maximum value at pH ranging between 3.6 and 4.8 (66% in this case), but was totally inactive at pH 5.8.

### Thermostability of Crude Extract Xylanase and Cellulase Activity

**FIGURE 5:**
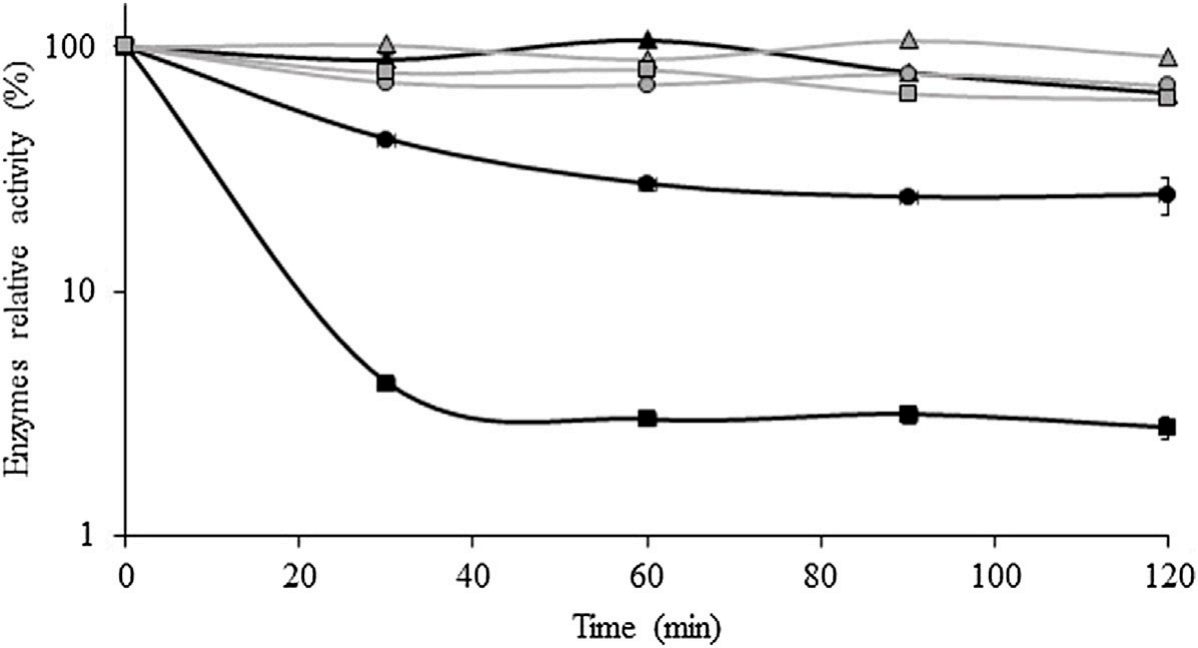
Thermal stability of xylanase and cellulase obtained after the solid-state fermentation of BSG at 45 °C (▲), 50 °C (•), and 60 °C (■). Xylanase and cellulase activities are represented by black and gray symbols, respectively. Values are presented as the mean ± standard deviation of two independent replicates.

Figure 5 shows the variation of xylanase and cellulase activities with the storage time at 45°C, 50°C, and 60°C. Cellulase was more thermostable than xylanase at all temperatures tested. For example, at 45°C, cellulase and xylanase maintained 91% and 64% of their activity, respectively, after 120 min of storage. Moreover, cellulase retained 69% and 61% of its initial activity after 2 h of storage at 50°C and 60°C, respectively. On the other hand, xylanase remained practically stable at 45°C, but at 50°C and 60°C, the activity decreased to 58% and 96% from its initial value, respectively, after 30 min.

### Enzymatic Hydrolysis of BSG_EF_

Response surface methodology was used to predict the crude extract quantity, BSG_EF_ load, and commercial β-glucosidase supplementation level that maximizes total phenol (TP) release, reducing sugars, and antioxidant activity (AA) during the enzymatic hydrolysis of BSG_EF_, within the range of values defined for each variable. The quantity of crude extract was determined based on the cellulase units due to its lower content and higher stability than xylanase in the crude extract at the enzymatic hydrolysis pH used. Commercial β-glucosidase addition was considered since its activity in the crude extract is low.

The matrix design, number of experiments performed, and increase of AA, TP, and sugar content released by enzymatic hydrolysis are presented in Table 2. In comparison with the control (without enzymatic treatment), the TP increase ranged from 8.2 to 49.3 mg GAE g^−1^ (run 4 and 6, respectively), the variation of AA ranged from −4.5 to 83.5 μmol TE g^−1^ (run 14 and 9, respectively), and the total sugars ranged from 9.76 to 65.5 mg g^−1^ (run 12 and 6, respectively).

**TABLE 2:** Matrix of the experimental design and identification of the different conditions applied in each experiment. Each variable and coded levels used in the Box–Behnken experimental design matrix are identified. Values are present as the mean [X_1_–crude extract; X_2_–load of BSG_EF_; X_3_–commercial β-glucosidase; TPV–total phenols variation; AAV–antioxidant activity variations; RS–released sugars; pentoses (xylose + arabinose)].

*Independent variables*				*Experimental values*						
Runs	X_1_	X_2_	X_3_	TPV (mg GAE g^−1^)	AAV (µmol TE g^−1^)	RS (mg g^−1^)		Glucose (mg g^−1^)		Pentoses (mg g^−1^)
*1*	1	0	−1	10.42 ± 0.50	16.06 ± 0.86	32.11 ± 1.61		17.03		15.08
*2*	0	0	0	8.61 ± 0.48	42.52 ± 8.84	35.26 ± 1.76		18.17		17.09
*3*	0	0	0	10.67 ± 0.72	42.36 ± 1.32	38.25 ± 1.91		19.54		18.7
*4*	−1	0	−1	8.18 ± 0.41	30.46 ± 2.20	16.82 ± 0.84		11.66		5.17
*5*	1	−1	0	18.92 ± 0.66	59.22 ± 13.77	30.82 ± 1.54		17.14		13.68
*6*	0	−1	−1	49.30 ± 0.25	28.79 ± 0.82	65.5 ± 3.06		27.43		33.83
*7*	1	0	1	12.19 ± 0.50	59.25 ± 3.45	18.99 ± 0.82		8.91		7.42
*8*	−1	0	1	15.11 ± 2.02	41.06 ± 12.83	18.64 ± 0.93		9.60		9.04
*9*	0	−1	1	42.87 ± 0.26	83.48 ± 5.61	34.13 ± 1.71		18.0		16.13
*10*	−1	−1	0	29.47 ± 2.96	56.83 ± 12.29	27.42 ± 1.37		13.72		13.71
*11*	0	0	0	11.04 ± 2.48	32.08 ± 3.29	36.8 ± 0.66		13.72		3.58
*12*	−1	1	0	8.89 ± 0.92	3.98 ± 1.61	9.76 ± 0.49		9.6		3.46
*13*	0	1	1	9.11 ± 0.32	0.81 ± 3.29	14.72 ± 0.74		7.5		4.86
*14*	1	1	0	8.76 ± 0.45	−4.54 ± 0.82	18.07 ± 0.9		12.0		6.07
*15*	0	1	−1	8.29 ± 0.63	−0.66 ± 1.41	21.2 ± 1.06		13.72		7.49

Independent variables					Identification	Coded levels				
						-1	0		1	
Crude extract (U cellulase g^−1^ of dry BSG_EF_)*					X_1_	50	100		150	
BSG_EF_ (% w/v)					X_2_	1	2.5		4	
Commercial β-glucosidase (U g^−1^ dry BSG_EF_)					X_3_	0	10		20	

* 50 U g^−1^ of cellulase activity corresponds to 165.1 U g^−1^ xylanase activity and 1.06 U g^−1^ of β-glucosidase.

The models for the three variables studied showed a good fit, with an adjusted *R* ^2^ above 0.95, which indicates that more than 95% of the variability of TP, AA, and reducing sugars is explained by the models (Table 3). The three models were statistically significant (*p* < 0.05), and the high F-value obtained (higher than F_tab_) also demonstrated the excellent fit of the three models.

**TABLE 3:** Quantitative model assessment tools (TPV—total phenol variation; AAV—antioxidant activity variations; RS—released sugars).

Tools	*Dependent variables*		
	TPV	AAV	RS
*R* ^2^	0.9981	0.9924	0.9981
*R* ^2^ adjusted	0.9924	0.9471	0.978
F-value	85.91	21.88	89.76
Significance level	98.84	95.55	98.89

**FIGURE 6:**
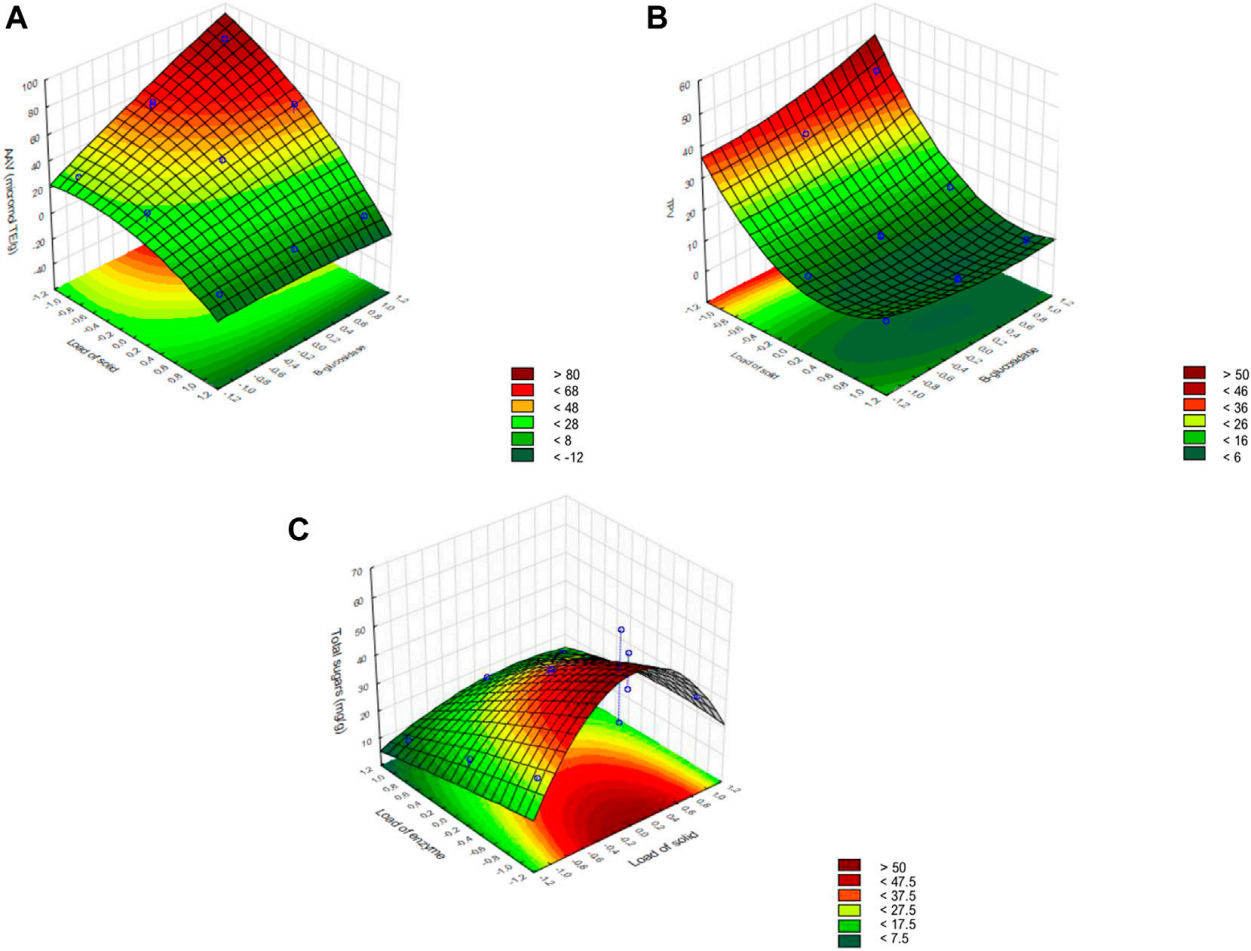
Surface response for the dependent variables: **(A)** antioxidant activity variation (AAV); **(B)** total phenols variation (TPV); and **(C)** total sugars.

Figure 6 shows each dependent variable in function of the two independent variables with a higher effect in the enzymatic hydrolysis, and the third independent variable fixed at the center level. The higher amount of AA and TP was released at the highest levels of the β-glucosidase addition and the lowest level of the BSG_EF_ load (Figures 6A,B). The quantity of crude extract did not affect the TP and AA release but had a high effect on reducing sugar release than the addition of β-glucosidase (Figure 6C).

The model predicted that the ideal conditions to maximize AA release (93.5 μmol TE g^−1^) during BSG_EF_ enzymatic hydrolysis, among the levels tested for each variable, are 150 U g^−1^ of cellulase from the crude extract, 1% (w/v) BSG_EF_, and 20 U g^−1^ β-glucosidase. To maximize TP release (40.9 mg g^−1^), the optimal conditions predicted by the model within the studied levels are 100 U g^−1^ of cellulase from the crude extract, 1% (w/v) BSG_EF_, and 20 U g^−1^ β-glucosidase. To maximize sugars yield (65.5 mg g^−1^), the optimal conditions within the levels tested in the present study predicted by the model are 50 U g^1^ of cellulase from crude extract, 2.5% (w/v) BSG_EF_, and no addition of β-glucosidase.

According to these results, the optimal conditions for enzymatic hydrolysis of BSG_EF_ were tested. Under these conditions, AA (89.5 ± 2.5 μmol TE g^−1^), TP (39.2 ± 1.3 mg g^−1^), and reducing sugars (65.5 mg g^−1^) release were similar to those predicted by the model, revealing that the model used was reasonably accurate. The sugar yield with these optimal conditions was 2.4 times higher than that achieved by the enzymatic hydrolysis of the unfermented BSG.

### Effect of Dietary Supplementation With Crude Extract in European Seabass Growth and Feed Efficiency

The crude extract dietary supplementation effect on growth and feed utilization of the European seabass is presented in Table 4. Growth performance, measured as final body weight, weight gain, and daily growth index, was not affected by the dietary treatments. However, feed efficiency and protein efficiency ratio of fish fed with the CE0.4 diet were higher than those of the control diet. Nitrogen (% nitrogen intake) and energy (kJ kg^−1^ ABW day^−1^) retention tend to be higher with the CE0.4 diet, but no significant differences were detected.

**TABLE 4:** Growth performance and feed utilization of the European seabass juveniles fed with the experimental diets for 64 days*.

Diets	Control	CE0.1	CE0.4	SEM
Initial body weight (g)	22.00	22.01	22.02	0.01
Final body weight (g)	35.08	35.67	36.78	0.51
Weight gain (g kg^−1^ ABW day^−1^)	8.95	9.29	9.84	0.27
Daily growth index	0.92	0.96	1.02	0.03
Feed intake (g kg^−1^ ABW day^−1^)	13.73	12.59	11.36	0.56
Feed efficiency	0.65^a^	0.74^a^ ^,^ ^b^	0.88^b^	0.04
Protein efficiency ratio	1.36^a^	1.54^a^ ^,^ ^b^	1.80^b^	0.08
Nitrogen retention (g kg^−1^ ABW day^−1^)	0.24	0.25	0.24	0.01
Nitrogen retention (% NI)	22.64	25.71	26.84	0.99
Energy retention (kJ kg^−1^ ABW day^−1^)	72.48	64.45	80.86	5.89
Energy retention (% EI)	22.02	21.24	29.14	1.87

*Values are presented as the mean (n = 3) and standard error of the mean (SEM). Values in the same row and different superscript letters are significantly different (Tukey’ test; *p* < 0.05).

Average body weight (ABW) = ((initial body weight, IBW + final body weight, FBW)/2).

Weight gain = (FBW-IBW ×1,000)/(ABW × time in days).

Daily growth index = (FBW^1/3^ - IBW^1/3^)/time in days) ×100.

Feed efficiency = wet weight gain/dry feed intake.

Protein efficiency ratio = wet weight gain/dry protein intake.

Nitrogen retention (g/kg ABW/day) = ((FBW×FBN − IBW×IBN) ×1,000)/(ABW × time in days); IBN and FBN: initial and final nitrogen content.

Energy retention (g/kg ABW/day) = ((FBW×FBE − IBW×IBE) ×1,000)/(ABW × time in days); IBE and FBN: initial and final energy content.

Energy retention (% energy intake) = whole-body energy retention/energy intake; EI) ×100.

## Discussion

### BSG_EF_ Production

The SSF of BSG resulted in solid matrix deconstruction leading to total mass solubilization of around 35%, mainly due to the remarkable reduction of hemicellulose and cellulose of 58% and 55%, respectively. Lignin was also reduced but to a smaller extent (42%), probably due to the inability of *A. ibericus* to produce lignin peroxidases. Opazo et al. (2012) carried out the SSF of soybean meal with cellulolytic bacteria and achieved 24% reduction in the NSP content, which compares well with the 28% reduction observed in the present study. The SSF of rice straw using *A. terreus* showed a reduction of 16% and 33% of cellulose and hemicellulose contents, respectively (Jahromi et al., 2011).

The SSF resulted in an absolute protein reduction of 25% in BSG, which may be due to the protein consumed by *A. ibericus* during the SSF (Gowthaman et al., 2001). Moreover, the protein decrease of BSG_EF_ can also be due to the extraction of soluble protein when aqueous extraction was carried out to obtain the crude extract at the end of the SSF. However, besides the absolute high protein decrease observed after SSF, the fermented BSG has a remarkably reduced lignocellulosic matrix and a considerable absolute protein content, which shows the nutritional and digestibility upgrade that the SSF exerted on BSG. Therefore, the fermented BSG could be a viable alternative to traditional feedstuffs commonly used in formulated feeds, such as in diets for aquatic animals (Dawood & Koshio, 2019).

### SSF Scale-Up

Successful upscaling processes of the SSF need the utilization of bioreactors with optimized designs that assure the maintenance and monitoring of ideal growing conditions of the fungi (Webb, 2017). This study has successfully scaled-up the SSF by 5-fold or 40-fold of BSG mass. Moreover, maximum enzymatic activities were observed using 50 g of BSG, showing that the solid bed-height increase from the flask to tray system did not affect the enzyme production. This better performance of the SSF in the tray-type system than that of the Erlenmeyer flask may be explained by the lower ratio of headspace to a total volume that reduces moisture loss. The further increase of BSG mass up to 400 g in trays resulted in a decrease of cellulase activity compared to the 50 g of BSG, despite the same bed-height. The production of this enzyme may be conditioned by inefficient substrate mixing, performed manually every day during the SSF, resulting in temperature increase and, concomitantly, compromising the production of the enzymes by the fungi (Webb, 2017). In the previous work, Sousa et al. (2018) observed that the SSF of BSG with *A. ibericus* resulted in the highest enzymatic activities among other agro-industrial substrates and fungi utilized, obtaining an extract with about 55 and 50 U g^−1^ of xylanase and cellulase activities, respectively. The SSF of 400 g of BSG with *A. brasiliensis* resulted in xylanase, cellulase, and β-glucosidase activities of 3,152, 7.3, and 19 U g^−1^ dry BSG, respectively (Outeiriño et al., 2019). The successful scale-up of the SSF of different agricultural by-products from flasks to tray-type bioreactors was also previously achieved for glucoamylase production by *A. niger* (Nahid et al., 2012). Furthermore, up-scaling from 24-g flasks to the 5.5-kg bioreactor of the SSF of a mixture of agro-industrial by-products with *Trichoderma asperellum* also increased the lipase, cellulase, and amylase production (Rayhane et al., 2019). Contrarily, scaling-up the SSF of wheat bran combined with sugarcane bagasse from 10 g up to 15 kg in a pilot packed-bed bioreactor led to a decrease of lipase activity by 57% (Pitol et al., 2017).

### Optimal Temperature and pH for Enzymes Activity

Cellulase and xylanase produced by *A. ibericus* during the SSF of BSG and present in the crude enzymatic extract were characterized regarding their activity with temperature and pH conditions and their thermostability. This characterization is fundamental to assess the optimal conditions for further utilization in diverse industrial processes.

In the present study, the optimum temperature that maximizes xylanase activity was determined to range between 45°C and 65°C, with maximum activity at 55°C. Similarly, maximum activity of xylanase produced by *Aspergillus flavus* was attained at 55°C (Chen et al., 2019), that of xylanase produced by *Simplicilium obclavatum* during the wheat bran SSF was attained at 50°C (Roy et al., 2013), that of xylanase produced by *Rhyzopus oryzae* during the SSF of raw oil palm frond leaves was attained at 60°C (Ezeilo et al., 2020), and that of xylanase produced by *A. niger* after 10 days of the SSF of BSG was attained at 50°C (Liguori et al., 2021). However, other authors reported lower optimal temperatures for xylanase activity, such as that produced from *Pediococcus acidilactici*, showing maximum activity at 40°C (Adiguzel et al., 2019). Cellulase activity was maximum at 50°C, but good activity levels were observed between 35°C and 65°C. Similarly, optimum cellulase activity was also observed at 50°C when fermenting raw oil palm leaves with *Rhizopus oryzae* (Ezeilo et al., 2020), at 55°C for the cellulase produced by *Trichoderma reesei* (Astolfi et al., 2019), and at 60°C for the cellulase produced by *A. niger* during the SSF of BSG (Liguori et al., 2021).

Regarding optimum pH, maximum xylanase activity was achieved at pH 4.4, while maximum cellulase activity was achieved at pH 3.9. Similarly, higher activities of cellulase and xylanase produced by *A. fumigatus* during the SSF of untreated oil palm trunk were observed at acid pH (4), while above the pH of 5, the enzymatic activities were dramatically reduced (Ang et al., 2013). In addition, *A. terreus* showed optimum cellulase activity in the pH range of 4–5 (Narra et al., 2014). Contrarily, the highest xylanase and cellulase activities obtained by the SSF of BSG with *A. niger* were found at neutral pH (Liguori et al., 2021).

The characterization of optimal temperature and pH of xylanase and cellulase in the crude enzymatic extract anticipates that these enzymes are suitable for converting different agro-industrial materials with different industrial applications. For example, these enzymes can be used in pulp, paper, food, and beverage industries as well to produce second-generation biofuels (Manisha & Yadav, 2017; Astolfi et al., 2019; Liguori et al., 2021), given their suitability to work at increasing temperatures and challenging pH levels.

### Thermostability of Crude Extract Xylanase and Cellulase

The thermostable enzymes have many advantages in the industrial and biorefinery processes of lignocellulosic materials. Generally, the enzymatic hydrolysis of lignocellulosic materials is carried out at temperatures ranging between 40°C and 50°C (Patel et al., 2019).

The present results showed that cellulase activity is more stable than xylanase, remaining practicably stable (less than 20% loss) for the first 60 min at all temperatures tested. On the other hand, xylanase activity had an identical profile to cellulase only at 45°C, showing significant activity loss as the temperature increased, being residual (around 4% of relative activity) after 30 min at 60°C. Delabona et al. (2013) also observed a decrease of xylanase and cellulase activities produced by *A. fumigatus* after 30 and 40 min at 60°C, respectively, although cellulase still maintained 60% of its activity after 1 h. Xylanase activity also showed less thermostability than cellulase in the enzymatic cocktail produced by *A. niger* during the SSF of sugarcane bagasse (Vasconcellos et al., 2015). In this study, the thermostability of endo-cellulases depended on the type of fermentation as enzymes produced by the SSF have shown higher stability than enzymes produced by the submerged fermentation (Vasconcellos et al., 2015).

### Enzymatic Hydrolysis of BSG_EF_ With Crude Extract

One of the aims of the present study was to develop a SSF-based biorefining strategy to maximize the release of reducing sugars and phenolic compounds from the hydrolysis of BSG_EF_. Pretreatment of lignocellulosic substrates for biorefineries, involving chemical and physical treatment, is a common procedure to increase the yield of different cell compounds (Socaci et al., 2018; Birsan et al., 2019; Alonso-Riaño et al., 2020). Biological pretreatment, such as SSF, is a new alternative strategy followed in this study. To maximize the release of AA, TP, and reducing sugars, the BSG_EF_ was subjected to the enzymatic hydrolysis with the crude extract.

The optimum enzymatic hydrolysis conditions were determined based on the Box–Behnken experimental design. The response surface methodology was highly accurate to identify each variable’s importance among the levels tested in the release of AA, TP, and reducing sugars from BSG_EF_, as the predicted values estimated by the model and the experimentally obtained values of TP, AA, and reducing sugars release were very similar, and the data showed a good fit to the model with high significance levels.

During the BSG_EF_ hydrolysis, among the tested levels for each variable, a low level of BSG_EF_ (1%) and β-glucosidase supplementation is required to increase the TP and AA release, while the crude extract quantity had no effect on TP and AA release. Thus, further increase of the enzymatic level (from crude enzymatic extract and β-glucosidase) may lead to higher TP and AA release from BSG_EF_ than the one obtained herein. The required supplementation with β-glucosidase may be attributed to the BSG_EF_ lignocellulosic structure that was previously modified during the SSF by the action of *A. ibericus*. Cellulose degradation by the integrated action of endo-glucanases, exo-glucanases, and β-glucosidase, and β-glucosidase acts in cellobiose hydrolysis (Lakhundi et al., 2015). The action of β-glucosidase results in the release of glucose (Lakhundi et al., 2015) and phenolic compounds linked to glycosides into their respective aglycones (Martins et al., 2011). Since the crude extract had low β-glucosidase activity (5.3 U g^−1^ BSG) compared to that of xylanase and cellulase (1,072.9 U g^−1^ BSG and 323.2 U g^−1^, respectively), the addition of a commercial β-glucosidase aimed to potentiate the release of phenolic compounds with antioxidant activity. Previously, Ajila et al. (2011) observed that phenolic compounds released from apple pomace were positively correlated with β-glucosidase, resulting in increased antioxidant activity. With other substrates, such as citrus by-products, β-glucosidase increased the release of TP and AA (Ruviaro et al., 2019). However, the enzymatic hydrolysis of BSG with β-glucosidase to extract phenolic compounds was not yet studied.

In the present work, the enzymatic hydrolysis of BSG_EF_, for 72 h, extracted 49.2 mg GAE g^−1^ BSG of TP, which was much higher than that obtained with other treatments, such as the SSF, alkaline and acid hydrolysis, ultrasounds, or organic solvents (Table 5). Enzymatic treatments allow releasing phenolic compounds linked to hemicellulose and lignin, which are not hydrolyzed by organic solvents (Alonso-Riaño et al., 2020) or which can be destroyed when in contact with strong acidic or alkaline conditions (Crowley et al., 2017).

**TABLE 5:** Phenolics yield obtained after different pretreatments of BSG reported in the present work and other studies.

Treatment	Conditions	Phenolic yield	References
SSF	2 g BSG, 7 days, 25°C, *A. niger*	2 mg GAE g^−1^	Leite et al. (2019)
Alkaline	2 g BSG, NaOH 2 M, 4 h, 65°C	16.2 mg GAE g^−1^	Alonso-Riaño et al. (2020)
Acidic	0.2 g BSG, methanol and Sulphur acid, 20 h, 85°C	30 mg GAE g^−1^	Alonso-Riaño et al. (2020)
Ultra-sounds	20 kHz, 1 h, 47°C	3.3 mg GAE g^−1^	Alonso-Riaño et al. (2020)
Organic solvents	2 g BSG, methanol, ethanol, or acetone, 30 min	1.2 mg GAE g^−1^	Socaci et al. (2018)
Enzymatic hydrolysis	10–100 µl carbohydrases g^−1^ dry BSG, 4 h or 9 h, 50°C	0.56 mg GAE g^−1^	Crowley et al. (2017)
Enzymatic hydrolysis	50 U cellulase g^−1^ dry BSG_EF_, 72 h, 45°C	49.2 mg GAE g^−1^	Present work

Alonso-Riaño et al. (2020) evaluated TP extraction from BSG with three xylanase levels, achieving maximum extraction of 42 mg GAE g^−1^ with the highest enzyme load. Using the commercial carbohydrases (10–100 µl carbohydrases g^−1^ dry BSG), 0.56 mg GAE g^−1^ of TP was obtained from BSG by Crowley et al. (2017), whereas in the present work, TP release from BSG reached 49.2 mg GAE g^−1^, highlighting the advantages of enzymatic hydrolysis of BSG after a biological pretreatment with the SSF.

By the enzymatic hydrolysis of BSG_EF_, the release of reducing sugars was highest when a low quantity of crude extract and an intermediate level of BSG_EF_ were used without the addition of commercial β-glucosidase. The low level of crude extract required to release reducing sugars from BSG_EF_ may be due to the previous partial disruption of BSG cell wall during the SSF before the enzymatic hydrolysis, which may have improved the enzymatic hydrolysis efficiency. Therefore, SSF worked as a pretreatment that potentiated the reducing sugars yield following the enzymatic hydrolysis. The importance of pretreatments to enhance the enzymatic hydrolysis efficiency of lignocellulosic materials has been pointed out (Paz et al., 2019). Llimós et al. (2020) observed that lignocellulosic enzymes produced during the SSF of BSG with *A. niger* released 0.56 g sugars per gram of dry BSG.

Other pretreatment procedures are often applied before the enzymatic hydrolysis, such as chemical pretreatments using diluted acids (Rojas-Chamorro et al., 2020). A combination of alkaline and ionic liquid pretreatments enhanced the sugar yield of sunflower stalk with hydrolyzed fungal carbohydrases (Nargotra et al., 2018). In addition, alkaline and ionic pretreatments combined with the fungal enzyme hydrolysis of BSG increased xylose release (Paz et al., 2019). Other novel pretreatments, as those using deep eutectic solvents, such as choline chloride–glycerol at 115°C, resulted in a yield of 160 mg glucose g^−1^ of pretreated BSG (Procentese et al., 2018). Biological pretreatments, such as SSF, have also been used. For example, Méndez-Hernández et al. (2019) subjected the corn stover to a biological pretreatment using a thermotolerant fungus, *Fomes* sp., and observed a 60% improvement of sugars released after 7 days in comparison to the untreated corn stover. The SSF followed by the enzymatic hydrolysis of a green macroalgae (*Ulva rigida*) with enzymes produced during the SSF increased the glucose yield by 53% (Fernandes et al., 2019). Outeiriño et al. (2019) reported that an enzymatic cocktail produced during the SSF of *A. brasiliensis* resulted in 44.8% and 21.5% saccharification of glucan and xylan, respectively. Given the promising yields following the application of biological treatments, the fungal pretreatment is an environmental-friendly procedure with great potential and efficiency in recovering reducing sugars by modifying the lignocellulosic structure of diverse agro-industrial by-products.

### Growth Performance

Diets for aquaculture fish, particularly those for the carnivorous species, rely on fish meal and agricultural ingredients such as soybean, corn gluten, and wheat. However, to ensure the long-term sustainability and profitability of aquaculture, the use of locally produced ingredients and agro-industrial by-products is of utmost importance (Oliva-Teles et al., 2015). The nutritional value of these alternative ingredients for aquaculture fish is generally low due to the reduced nutrient digestibility and the presence of anti-nutritional factors, such as NSPs (Daniel, 2018), which can impair growth, feed utilization, and fish welfare and health (Gatlin et al., 2007; Xavier et al., 2012).

To counteract the adverse effects of plant-feedstuff NSP, technological strategies have been developed, such as pretreatment of plant feedstuffs and diet supplementation with carbohydrases (Castillo & Gatlin, 2015). The use of carbohydrases to increase the digestibility of plant-based diets is a promising strategy for aquaculture since fish have limited capacity to utilize dietary carbohydrates (Sinha et al., 2011). In the present study, the crude extract was lyophilized to allow its incorporation in the diets, but other methods of concentration can be considered in a scaled-up process, such as alternative lower power-consuming methods that are already in use at the industrial level. The inclusion of the crude extract linearly increased the feed efficiency and protein efficiency ratio (R = 0.81, *p* < 0.01; R = 0.79, *p* < 0.01, respectively). These results may be attributed to an increase in dietary carbohydrate availability due to the NSP hydrolysis by the action of the crude extract carbohydrases. Other authors also observed the beneficial effects of dietary supplementation with exogenous carbohydrases on feed utilization in different fish species (Magalhães et al., 2016; Diógenes et al., 2018; Maas et al., 2019). Maas et al. (2019) observed that a plant-based diet supplemented with an enzymatic cocktail of xylanase and phytase improved the growth and feed utilization of Nile tilapia (*Oreochromis niloticus*) after 6 weeks of feeding. Magalhães et al. (2016) observed an increase in feed utilization in white seabream (*Diplodus sargus*) fed with a diet supplemented with commercial nonstarch carbohydrases. Other studies also included enzymes obtained by the SSF in plant feedstuff-rich diets with positive results regarding nutrient utilization and growth performance in Nile tilapia (Novelli et al., 2017; Bowyer et al., 2020) and common carp (Anwar et al., 2020). In addition to the direct action upon the NSPs, dietary carbohydrase supplementation may also reduce digestive viscosity and, concomitantly, favor the access and time of action of the endogenous fish enzymes, increasing feed and protein utilization (revised by Castillo & Gatlin, 2015). For example, in turbot (*Scophthalmus maximus*), dietary supplementation with carbohydrases increased the amylase and lipase activity, whereas the total protease activity was not affected. In hybrid tilapia, dietary carbohydrases supplementation increased amylase but not protease or lipase activities (Li et al., 2009), while in white seabream only amylase activity was increased (Magalhães et al., 2018). Further studies are required to study the effect of dietary supplementation with the crude extract on the endogenous digestive enzymes activity.

The dietary supplementation with the crude extract increased feed efficiency, that is, decreased the amount of feed required per unit of fish produced. In aquaculture production, increasing feed efficiency is the most important feature to increase the production efficiency and economic profit and reduce environmental impacts (Besson et al., 2017) as more than 60% of the total aquaculture costs are associated with feed costs (Troell et al., 2014; Daniel, 2018). In addition, production of feed enzymes from the SSF of agro-industrial materials, such as BSG, also represent a probability towards the reduction of production costs and sustainability of enzyme industry (Lizardi-Jiménez & Hernández-Martínez, 2017) and contributes to the reduction of lignocellulosic biomass disposal in the environment (Sakhuja et al., 2021).

## Conclusion

A SSF-based biorefining strategy was applied to convert BSG into two distinct products: a protein-enriched fermented BSG with a modified lignocellulosic structure and a functional crude extract with highly active carbohydrases. BSG was fermented with *Aspergillus ibericus* to produce a crude extract to be used to further hydrolyze the fermented BSG or applied as a feed enzyme supplement in aquafeeds.

According to the Box–Behnken response surface methodology, the load of crude extract is a fundamental parameter to maximize the release of reducing sugars from BSG_EF_, while both the load of BSG_EF_ and β-glucosidase addition are key variables to maximize the release of total phenolics and antioxidant activity.

Potential application of this crude extract in aquafeeds was demonstrated in the European seabass fed with high plant-based diets, where a positive effect was confirmed in terms of feed and protein utilization.

Future work must be carried out to assess the feasibility of including the nutritionally enhanced fermented BSG in diets and test if higher inclusion levels of the crude extract can further improve feed utilization and growth of the European seabass.

## Data Availability

The raw data supporting the conclusions of this article will be made available by the authors, without undue reservation.
